# Neuropeptide Y-Induced Orexigenic Action Is Attenuated by the Orexin Receptor Antagonist in Bullfrog Larvae

**DOI:** 10.3389/fnins.2017.00176

**Published:** 2017-04-04

**Authors:** Kouhei Matsuda, Kairi Matsumura, Syun-suke Shimizu, Tomoya Nakamachi, Norifumi Konno

**Affiliations:** ^1^Laboratory of Regulatory Biology, Graduate School of Science and Engineering, University of ToyamaToyama, Japan; ^2^Laboratory of Regulatory Biology, Graduate School of Innovative Life Sciences, University of ToyamaToyama, Japan

**Keywords:** bullfrog larvae, NPY, orexin A, orexigenic action, antagonist, neuronal interaction

## Abstract

In bullfrog larvae at the pre- and pro-metamorphic stages, feeding behavior is regulated by appetite factors such as orexigenic peptides. In fact, food intake is enhanced by intracerebroventricular (ICV) administration of neuropeptide Y (NPY) and orexin A. Using goldfish, our previous study indicated that the orexigenic action of NPY is mediated by orexin A, suggesting the functional interaction between the two. However, there is little information about whether the action of orexin A mediates the orexigenic action of NPY in bullfrog larvae. Therefore, we examined the effect of the orexin receptor antagonist, SB334867 on the orexigenic action of NPY in larvae. The stimulatory effect of ICV injection of NPY at 10 pmol/g body weight (BW) on food intake was abolished by treatment with SB334867 at 60 pmol/g BW. These results suggest that, in bullfrog larvae, there is a neuronal relationship between the NPY and orexin systems, and that the orexigenic action of NPY is mediated by the orexin A-induced orexigenic action.

## Introduction

Neuropeptide Y (NPY) was first identified from porcine central nervous system. NPY is belonging NPY family of peptides including peptide YY and pancreatic polypeptide, and it is one of the most highly conserved neuropeptides in vertebrates and even in invertebrates (Tatemoto et al., 1982; Blomqvist et al., 1992). NPY occurs abundantly in not only the central, but also peripheral nervous systems, and is involved in many roles such as cardiovascular control and neuroendocrine function in mammals (Karl and Herzog, 2007). NPY stimulates feeding behavior, and it has been considered a potent orectic neuropeptide in the mammalian brain (Woods et al., 1998). Orexin is a neuropeptide that was first isolated as an orphan receptor ligand, and subsequently identified as an appetite stimulator (Sakurai et al., 1998). Orexin has two molecular forms, orexin A and B, derived from the same precursor. In addition to its orexigenic action, orexin regulates energy consumption, rhythmicity, and neuronal apoptosis (Matsuki and Sakurai, 2008; Shioda et al., 2008).

An understanding of the evolutionary process of the NPY- and orexin-regulated system of feeding behavior could provide discernment into the physiological role of these functions throughout the vertebrates. In anuran brains, the neuroanatomical distributions of NPY and orexin have been described in detail (Danger et al., 1985; Cailliez et al., 1987; Galas et al., 2001), and primary structures of frog NPY and *Xenopus* orexin have been determined (Chartrel et al., 1991; Shibahara et al., 1999). Our previous studies have indicated that, in bullfrog larvae at the pre- and pro-metamorphic stages, intracerebroventricular (ICV) administration of NPY and orexin A enhances food intake, suggesting that these neuropeptides act as potent orexigenic factors in the larvae of anuran amphibians (Shimizu et al., 2013, 2014b). Some previous papers have also documented the regulation of feeding by NPY and orexin A in fish (Lin et al., 2000; de Pedro and Bjornsson, 2001; Volkoff and Peter, 2001; Aldegunde and Mancebo, 2006; Gorissen et al., 2006; Matsuda and Maruyama, 2007; Xu and Volkoff, 2007; Matsuda, 2009). Especially, it has been shown that NPY and orexin A act as representative appetite enhancers in goldfish (López-Pati-o et al., 1999; Volkoff et al., 1999; Narnaware et al., 2000; Miura et al., 2006, 2007; Nakamachi et al., 2006). A previous study has also provided evidence that, in goldfish, NPY-induced appetite is mediated by orexin A, suggesting that the two peptides share a functional relationship for regulation of feeding (Volkoff and Peter, 2001). However, there is little information about the relationship between NPY and orexin A in the regulation of food intake in bullfrog larvae. Therefore, to clarify whether orexin A mediates the orexigenic action of NPY, we investigated the effect of ICV administration of an orexin receptor antagonist, SB334867, on the orexigenic action of ICV-injected NPY in bullfrog larvae.

## Materials and methods

### Animals

Bullfrog (*Rana catesbeiana*) larvae weighing 5–7 g were collected from ponds in the suburbs of Toyama City, Japan. The developmental stages of the larvae were determined according to Taylor and Kollros (1946). Since the body size of obtained larvae at premetamorphic stages was very small, experiments were done using 100 larvae at prometamorphic stages (XI–XIX). As previously described (Shimizu et al., 2013), the animals were kept for 1–2 weeks under controlled light/dark conditions (12L/12D) with the water temperature maintained at 20–24°C. The larvae were fed every day at noon with a powder diet (Itosui Co., Tokyo, Japan) until used in experiments. Animal experiments were conducted in accordance with the Invasive Alien Species Act of Japan and the University of Toyama's guidelines for the care and use of alien and laboratory animals, and were done by approval of an ethics committee of the University of Toyama and permission of government authorities (No. 05000361).

### Chemicals

In order to examine the effects of ICV administration of NPY and orexin A on food intake, and the effects of NPY receptor and orexin receptor antagonists on the actions of ICV-injected NPY and orexin A in prometamorphic larvae, the following chemicals were used. A NPY Y1-receptor antagonist, BIBP3226 (diphenylacetyl-D-Arg-4-hydroxybenzylamide, Bachem AG, Bubendorf, Switzerland) and a selective orexin receptor antagonist, SB334867 [*N*-(2-methyl-6-benzoxazolyl)-*N*′-1,5-naphthyridin-4-yl urea, Tocris Cookson Ltd., Bristol, UK], were purchased commercially, dissolved in dimethyl sulfoxide at 20–50 mM for storage, and diluted with 0.6% NaCl and 0.02% Na_2_CO_3_ solution (saline) before use. NPY (rat NPY, Peptide Institute Co., Osaka, Japan) and orexin-A (rat orexin-A, Peptide Institute) were also purchased commercially, dissolved in saline at 0.1 mM for storage at −80°C, and diluted with saline before use. 3-Aminobenzoic acid ethyl ester (MS-222) for anesthesia was obtained commercially from Sigma-Aldrich (St. Louis, MO, USA), and was prepared at concentration of 0.15%. Each animal was exposed to this solution for 2 min.

### Measurement of food intake

Details of the methods used for measuring food consumption in the larvae have been reported elsewhere (Matsuda et al., 2010; Shimizu et al., 2013, 2014a,b). As previously described (Shimizu et al., 2014b), two types of powder diet colored green and red, respectively (containing the same components as those described above), were obtained from Itosui Co., Tokyo, Japan. First, the test prometamorphic larvae were fed the green-colored diet and kept under laboratory conditions. Then, after a 24-h fast, an adequate amount of the red-colored food was made available at 3% of BW. Test substances were ICV-administered as described below, and after 15 min, each animal was decapitated and the gastrointestinal tract was removed. In our previous study, we observed food intake in intact and ICV-injected larvae during 60 min after recovery from anesthesia. Then, we determined that food intake is measured during first 15 min. The wet weight of the red-colored gastrointestinal contents was measured after removal of intestinal juice with tissue paper, and expressed as micrograms of food taken per gram BW. The experiments were conducted around noon.

### Effect of ICV administration of BIBP3226 and SB334867 on the orexigenic actions of ICV-injected NPY and orexin A

In order to double-check the orexigenic actions of NPY and orexin A, and the antagonistic actions of BIBP3226 and SB334867 upon them (Shimizu et al., 2013, 2014b), we examined the effects of ICV administration of NPY, orexin A, BIBP3226, and SB334867 on food intake in prometamorphic larvae. Our previous studies have indicated that simultaneous injection of antagonists with peptides induces their antagonistic effects (Shimizu et al., 2013, 2014b).

We did not have an atlas for bullfrog larval brain. However, according to our previous experiments, same procedures have been done. Therefore, following sentences were mainly quoted from our previous paper (Matsuda et al., 2010; Shimizu et al., 2013, 2014b). “For ICV administration of test substances, each animal was placed in a stereotaxic apparatus under anesthesia with MS-222. A small area (~1 mm^2^ square) of the parietal skull was carefully removed using a surgical blade (No. 19, Futaba, Tokyo, Japan), and then 0.1 μl/g BW (0.5–0.7 μl) of each test substance including Evans blue dye was injected into the third ventricle of the brain using a 10-μl Hamilton syringe with a 0.1-μl scale. The gap in the parietal skull was then filled with a surgical bonding agent (Aron Alpha, Sankyo, Japan). The accuracy of the injection site and volume was confirmed after the experiment by examining whether Evans blue dye was present in the ventricle without leakage. Control larvae in each experiment were injected with the same volume of saline in the same way as for the experimental group. Each larva that had received the ICV injection was placed individually in a small experimental tank (diameter 11 cm) containing 700 ml of tap water. After recovery from anesthesia, each larva was supplied with the red-colored food equivalent to 3% of its BW. After 15 min, the weight of the intestinal contents was measured as described above.”

“In order to check the effect of ICV injection of BIBP3226 on the orexigenic action of NPY at 10 pmol/g BW, half a microliter of BIBP3226 at 100 pmol/g BW was injected into the third ventricle of the brain of larvae as described above. The ICV-injected dose of BIBP3226 had been determined in previous experiments (Shimizu et al., 2013). Larvae in the control group were given injections of the same volume of saline-diluted dimethyl sulfoxide (vehicle). Following the administration of BIBP3226 or vehicle, either NPY at 10 pmol/g BW or saline was delivered by ICV injection. The feeding experiment was performed according to the procedures described above. In order to check the effect of ICV injection of SB334867 on the central actions of orexin A, 0.5 μl of SB334867 at 60 pmol/g BW in addition to orexin A at 6 pmol/g BW was injected into the third ventricle of the brain of the larvae. The ICV-injected dose of SB334867 had been determined by reference to a previous study (Shimizu et al., 2014b). Larvae in the control group were given an injection of the same volume of dimethyl sulfoxide diluted with saline.”

### Effect of ICV administration of SB334867 on the orexigenic action of ICV-injected NPY

According to our previous experiments, same procedures have been done. Therefore, following sentences were mainly quoted from our previous paper (Shimizu et al., 2014b). “In order to examine the effect of ICV injection of SB334867 on the central action of NPY, 0.5 μl of SB334867 at 60 pmol/g BW in addition to NPY at 10 pmol/g BW was injected into the third ventricle of the larval brain. Larvae in the control group were given an injection of the same volume of dimethyl sulfoxide diluted with saline. The feeding experiment was performed according to the procedures described above.”

### Data analysis

According to our previous experiments, same statistical analysis have been done. Therefore, following sentences were quoted from our previous paper (Kojima et al., 2009). “All results pertaining to the effect of receptor antagonists on food intake are expressed as the mean ± SEM. Statistical analysis was performed using two-way ANOVA with Bonferroni's method. Statistical significance was determined at the 5% level.”

## Results

### Effect of ICV injection of BIBP3226 on the orexigenic action of ICV-injected NPY

**Figure 1:**
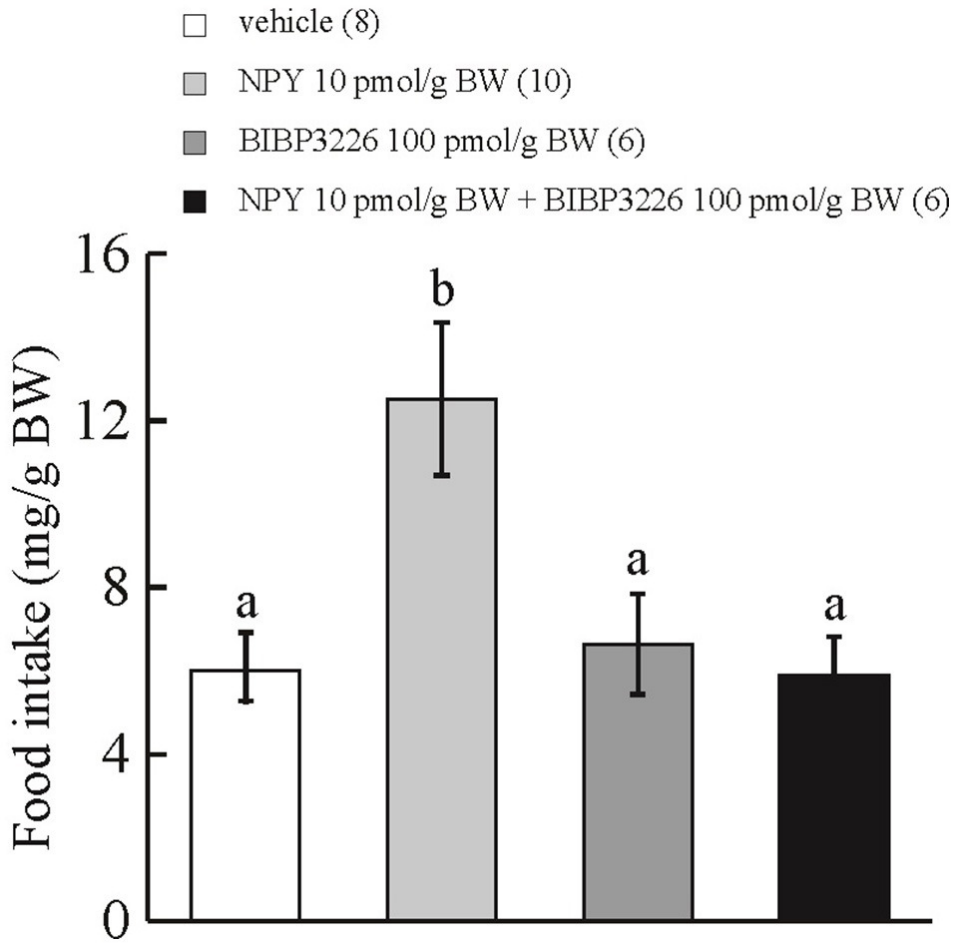
**Effect of ICV administration of a NPY Y1-receptor antagonist, BIBP3226, on the orexigenic action of NPY in prometamorphic larvae**. Each column and bar represents the mean and SEM, respectively, and the numbers in parentheses in the panels indicate the number of larvae in each group. Significance of differences was evaluated by two-way ANOVA with Bonferroni's method. Different superscripts (a and b) indicate statistical significance (*P* < 0.05).

ICV administration of NPY at 10 pmol/g BW stimulated cumulative food intake during the 15-min observation period in comparison with ICV injection of vehicle or BIBP3226 at 100 pmol/g BW. ICV injection of NPY at 10 pmol/g BW plus BIBP3226 at 100 pmol/g BW did not affect cumulative food intake during the 15-min observation period in comparison with ICV injection of vehicle (Figure 1). Interaction between treatments with NPY and BIBP3226 was significant by two-way ANOVA with Bonferroni's method (*F* = 5.83 and *p* = 0.023).

### Effect of ICV injection of SB334867 on the orexigenic actions of ICV-injected orexin A

**Figure 2:**
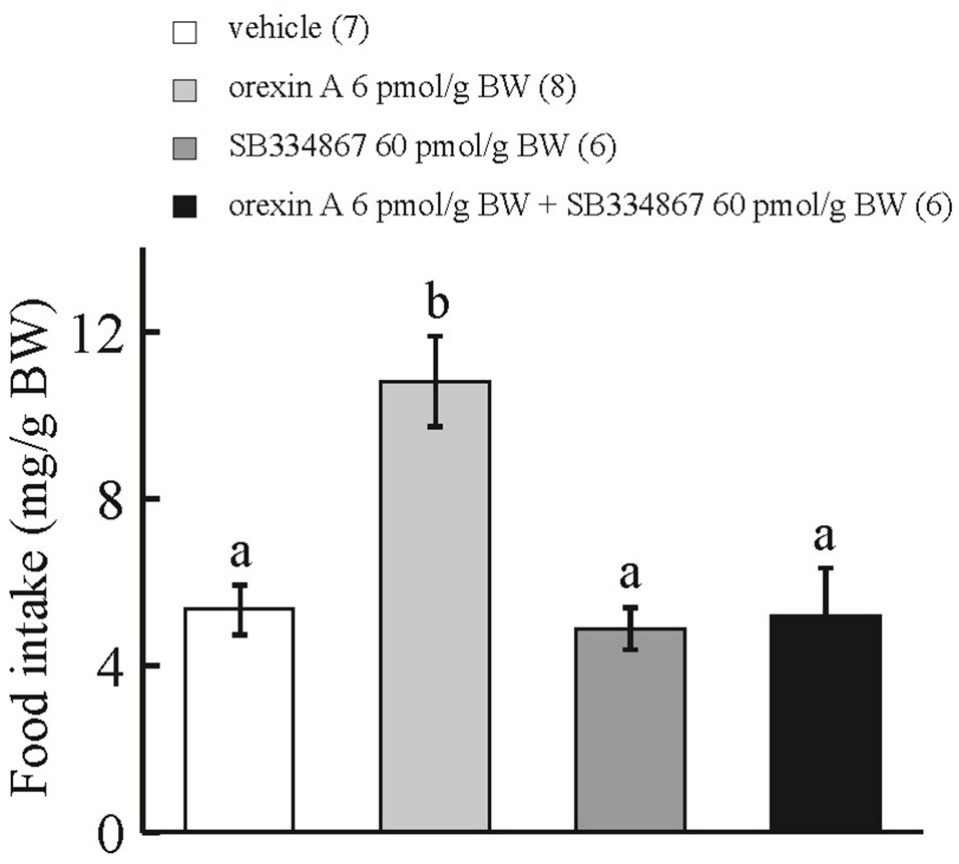
**Effect of ICV administration of an orexin receptor antagonist, SB334867, on the orexigenic action of orexin A in prometamorphic larvae**. Each column and bar represents the mean and SEM, respectively, and the numbers in parentheses in the panels indicate the number of larvae in each group. Significance of differences was evaluated by two-way ANOVA with Bonferroni's method. Different superscripts (a and b) indicate statistical significance (*P* < 0.05).

ICV administration of orexin A at 6 pmol/g BW enhanced cumulative food intake during the 15-min observation period in comparison with ICV injection of vehicle or SB334867 at 60 pmol/g BW. ICV injection of orexin A at 6 pmol/g BW plus SB334867 at 60 pmol/g BW did not affect cumulative food intake for 15 min after feeding in comparison with ICV injection of vehicle (Figure 2). Interaction between treatments with SB334867 and orexin A was significant by two-way ANOVA with Bonferroni's method (*F* = 8.05 and *p* = 0.009).

### Effect of ICV administration of SB334867 on the orexigenic action of ICV-injected NPY

**Figure 3:**
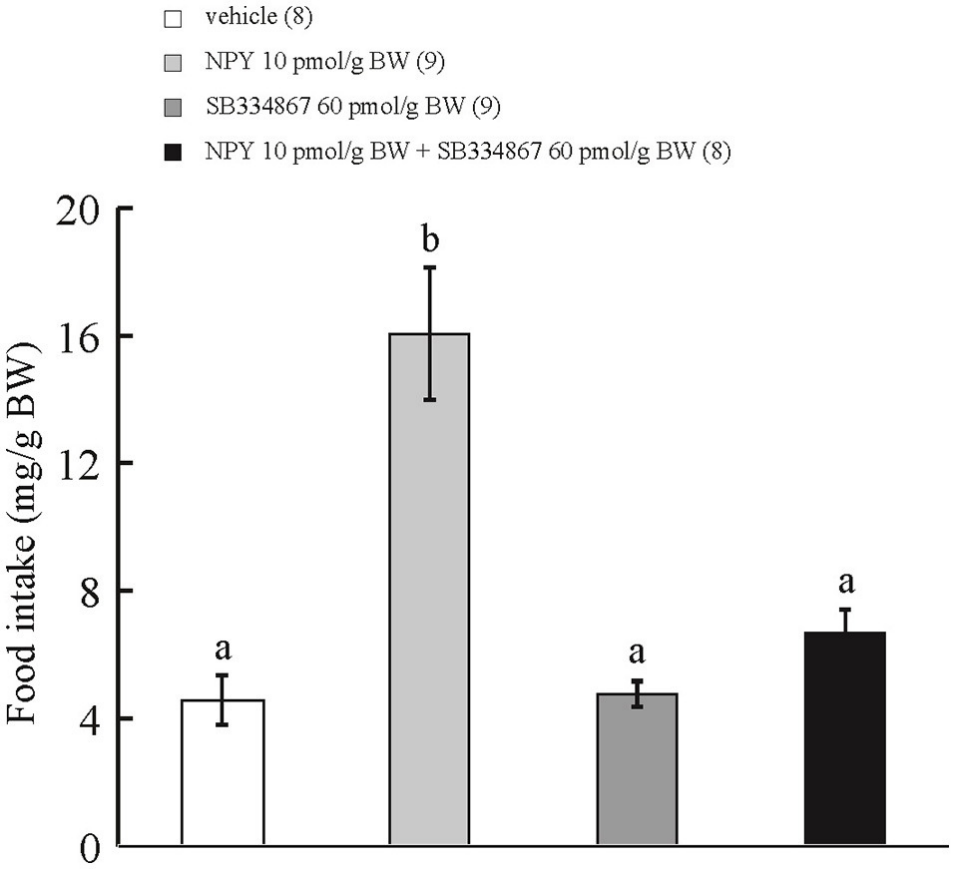
**Effect of ICV administration of SB334867 on the orexigenic action of NPY in prometamorphic larvae**. Each column and bar represents the mean and SEM, respectively, and the numbers in parentheses in the panel indicate the number of larvae in each group. Significance of differences was evaluated by two-way ANOVA with Bonferroni's method. Different superscripts (a and b) indicate statistical significance (*P* < 0.05).

ICV administration of NPY at 10 pmol/g BW increased cumulative food intake during the 15-min observation period in comparison with ICV injection of vehicle or SB334867 at 60 pmol/g BW. ICV injection of NPY at 10 pmol/g BW plus SB334867 at 60 pmol/g BW did not affect cumulative food intake during the 15-min observation period in comparison with ICV injection of vehicle (Figure 3). Interaction between treatments with SB334867 and NPY was significant by two-way ANOVA with Bonferroni's method (*F* = 31.9 and *p* = 0.0001).

## Discussion

Larvae of anuran amphibians can feed and grow until the metamorphic climax, because as metamorphosis progresses, many body parts such as the oral and digestive organs are reconstructed (Ishizuya-Oka and Shi, 2007; Wright et al., 2011). Therefore, feeding behavior in anuran larvae seems to be regulated by appetite and satiety factors during the pre- and pro-metamorphic periods. In bullfrog larvae, ICV administration of NPY, orexin A, or ghrelin induces an increase of food consumption, whereas ICV injection of corticotropin-releasing factor attenuates food intake (Matsuda et al., 2010; Shimizu et al., 2013, 2014a,b). These findings suggest that NPY, orexin A, and ghrelin are implicated in the regulation of feeding behavior as potent orexigenic neuropeptides in the larval brain. We obtained larvae at pre- and pro-metamorphic stages. However, premetamorphic larvae were very small. In the present study, we used prometamorphic bullfrog larvae, and double-checked the orexigenic actions of NPY and orexin A, and the antagonistic effects of BIBP3226 and SB334867 upon them.

In the goldfish model, our and other previous studies have indicated that the orexigenic action of NPY is mediated by the orexin A signaling pathway (Volkoff and Peter, 2001; Kojima et al., 2009): NPY-induced action was blocked by treatment with SB334867, and it was also attenuated by treatment with an excess amount of orexin A. Using a double-immunostaining and confocal laser scanning microscopy, neuroanatomical observation has indicated that neurons with NPY- and orexin-like immunoreactivities are located in the hypothalamic region, the nucleus posterioris periventricularis, in close proximity to each other neuron (Kojima et al., 2009). Accordingly, in this species, there seems to be a functional relationship between NPY and orexin A in the regulation of feeding. In the present study, we indicated for the first time that the action of NPY is blocked by treatment with SB334867. The results suggest that the orexigenic action of NPY is mediated via the orexin receptor in bullfrog larvae as well as in goldfish. Thus, it is likely that NPY and orexin A exert orexigenic actions in bullfrog larvae. This observation also supports the role of a functional relationship between NPY and orexin A in feeding regulation among vertebrates (Yamanaka et al., 2000; Niimi et al., 2001; Sahu, 2002). Bullfrog larvae can feed and grow until the metamorphic climax, and orexigenic factors such as NPY and orexin A seem to stimulate appetite, and to regulate energy balance of larvae during the developmental stages (Shimizu et al., 2013, 2014b). The present study suggests that NPY and orexin A is mutually involved in appetite regulation during the prometamorphic stages.

In conclusion, our data indicate that, in bullfrog larvae, the orexigenic action of NPY is mediated by the orexin A-induced orexigenic action.

## Author contributions

Conceived and designed the experiments: KoM, TN, and NK; performed the experiments: KaM and SS; analyzed the data: KoM and KaM; contributed materials: KaM and SS; wrote the paper: KoM; revising the paper: TN and NK.

### Conflict of interest statement

The authors declare that the research was conducted in the absence of any commercial or financial relationships that could be construed as a potential conflict of interest. The reviewer LG and handling Editor declared their shared affiliation, and the handling Editor states that the process nevertheless met the standards of a fair and objective review.
